# Antimicrobial resistance genes in microbiota associated with sediments and water from the Akaki river in Ethiopia

**DOI:** 10.1007/s11356-022-20684-2

**Published:** 2022-05-18

**Authors:** Berhanu Yitayew, Yimtubezinash Woldeamanuel, Daniel Asrat, Aminur Rahman, Adane Mihret, Abraham Aseffa, Per-Erik Olsson, Jana Jass

**Affiliations:** 1 College of Health Sciences, School of Medicine, Addis Ababa University, Addis Ababa, Ethiopia; 2 School of Science and Technology, The Life Science Center - Biology, Örebro University, 701 82 Örebro, Sweden; 3 Armauer Hansen Research Institute, Addis Ababa, Ethiopia

**Keywords:** Aquatic sediments, Antimicrobial resistance, Antimicrobial resistance genes, Urban water, β-lactamase genes, ESBL, Carbapenemase

## Abstract

The spread of antimicrobial-resistant pathogens is a global health concern. Most studies report high levels of antimicrobial resistance genes (ARGs) in the aquatic environment; however, levels associated with sediments are limited. This study aimed to investigate the distribution of ARGs in the sediments and water of the Akaki river in Addis Ababa, Ethiopia. The diversity and abundance of 84 ARGs and 116 clinically important bacteria were evaluated from the sediments and water collected from five sites in the Akaki river. Most of the ARGs were found in the city close to anthropogenic activities. Water samples collected in the middle catchment of the river contained 71–75% of targeted ARGs, with genes encoding aminoglycoside acetyltransferase (*aac(6)-Ib-cr*), aminoglycoside adenylyl transferase (*aadA1*), β-lactamase (*bla*_OXA-10_)_*,*_ quinolone resistance S (*qnrS*), macrolide efflux protein A (*mefA*), and tetracycline resistance (*tetA*), were detected at all sampling sites. Much fewer ARGs were detected in all sediments, and those near the hospitals had the highest diversity and level. Despite the lower levels and diversity, there were no unique ARGs detected in the sediments that were also not detected in the waters. A wide range of clinically relevant pathogens were also detected in the Akaki river. The findings suggest that the water phase, rather than the sediments in the Akaki river, is a potential conduit for the spread of ARGs and antibiotic-resistant bacteria.

**Supplementary Information:**

The online version contains supplementary material available at 10.1007/s11356-022-20684-2.

## Introduction

Decades of extensive antibiotic use to treat human and animal infections, and as food supplements in livestock (Bacanl and Başaran 2019), have led to the rapid emergence of antimicrobial-resistant bacteria (ARB) (Leung et al. 2011). Infectious diseases caused by ARB are again a major global public health problem, resulting in fewer treatment options for certain infections. Consequently, the morbidity and mortality due to infections caused by ARB is the biggest healthcare crisis of the twenty-first century (Fair and Tor 2014). According to the European Centre for Disease Prevention and Control, approximately 33,000 people die annually due to infections caused by ARB in Europe and the European Economic Area (Cassini et al. 2019). In Sub-Saharan Africa, the management of drug-resistant infections is exacerbated by the lack of diagnostic facilities and poor monitoring systems (Kariuki and Dougan 2014). To control the spread of ARB, research predominantly focused on isolates from clinical settings with little concern for environmental transmission (Martinez 2009). However, environments outside the healthcare facilities play a significant role in the spread of ARB (Kraemer et al. 2019). Antimicrobial resistance cannot be addressed by simply managing the problem in healthcare facilities; therefore it must be confronted from a One Health perspective across human, veterinary, and environmental boundaries (Wang et al. 2018).

Antimicrobial-resistant pathogens persist in the environment due to a combination of continued discharge of both fecal bacteria and antibiotic residues (Munir et al. 2011; Karkman et al. 2019). Most antibiotics are excreted in their active form, and despite some having a short half-life of less than one day (e.g., amoxicillin) (Braschi et al. 2013) in the environment depending on the conditions, continual discharge results in pseudo-persistent sub-inhibitory concentrations that select for ARB (Gullberg et al. 2011; Munir et al. 2011). However, some antibiotics such as tetracyclines and macrolides have a much longer half-life in water but even longer in the sediment (Harrower et al. 2021). This is relevant since sediments in aquatic systems are considered an important reservoir for antimicrobial resistance genes (ARGs) and facilitate the dissemination of antimicrobial-resistant pathogens in the environment (Zhu et al. 2017). ARGs associated with the aquatic sediments are believed to be persistent and thus may continuously be released into the associated water compartment (Luo et al. 2010). Therefore, understanding the diversity of ARGs in the sediment and water phase is important for sustainable antimicrobial resistance control.

The negative impact of human activities along aquatic environments is a concern for the persistent spread of antimicrobial-resistant pathogens (Jiang et al. 2018). Bacteria can acquire antibiotic resistance through mutations and horizontal gene transfer, and this not only contributes to the abundance and diversity of resistance genes in aquatic environments but also distributes them among the different bacteria (Smillie et al. 2011). However, a major source of ARB in the aquatic environment is the release of untreated effluent from healthcare facilities and households (Kraemer et al. 2019). Human and animal gut microbiota contain a wide range of ARGs, thus sewage discharge and fecal contamination has been linked to the increased abundance of ARGs in the aquatic environment (Karkman et al. 2019). It is also noteworthy that there are naturally resistant environmental bacteria that also contribute to the total abundance of ARB in the aquatic environment (Allen et al. 2010).

In recent years, increasing levels of ARGs have been reported in aquatic environments (Zhou et al. 2017). Most of these studies have used culture-based methods commonly involving the isolation of target bacteria on general or selective media followed by assessing for ARGs. Evaluating antibiotic resistance in culturable bacteria from the environment will only provide partial information and will miss detecting ARGs in bacteria that are non-culturable. Therefore, investigating ARGs in bacterial communities provides a comprehensive view of antibiotic resistance in the environment. In the current study, we explored the diversity and abundance of ARGs and clinically relevant bacteria in the sediment and associated water using a high-throughput DNA qPCR analysis for 84 major genes associated with resistance to β-lactams, aminoglycosides, fluoroquinolones, macrolides, vancomycin, and erythromycin. The community of clinically relevant bacteria was also determined in the same sample. To the best of our knowledge, this is the first study to investigate ARGs in bacterial communities in the Akaki river system of Ethiopia.

## Materials and methods

### Description of study area and sampling sites

**Fig. 1:**
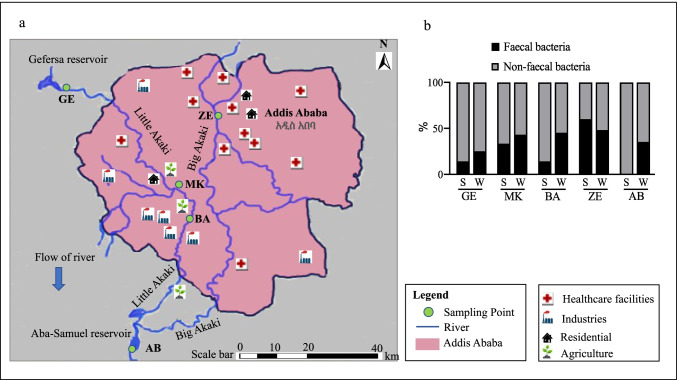
**a** The map of Little Akaki and Big Akaki river system flowing through Addis Ababa, Ethiopia. The Little Akaki River originates in the north-western part of Addis Ababa, while the Big Akaki River originates in the north-eastern region of the city. The sampling points include Gefersa (GE), Mekanissa (MK), and Batu (BA) from Little Akaki river and Zewditu (ZE) from Big Akaki river. Both rivers converge downstream in the Aba-Samuel (AB) reservoir. GE is upstream of the Little Akaki river and serves as a drinking water reservoir (9°03′43.2″N 38°38′34.9″E), MK is located in irrigation and residential area (8°58′25.7″N38°43′59.6″E), BA is located in an industry-dominated area and irrigation zone (8°55′52.0″N 38°45′26.3″E), ZE is located along residential and healthcare facilities (9°01′02.9″N 38°45′20.2″E), and AB is a downstream reservoir (8°47′15.7″N 38°42′25.7″E). The estimated distance between sampling sites is 16.85 km (GE-MK), 8.3 km (MK-BA), 25.3 km (BA-AB), and 52.16 km (ZE-AB). **b** Proportion of fecal and non-fecal bacteria detected by PCR in the Akaki river sediments (S) and water (W)

The study was conducted in Addis Ababa, the capital city of Ethiopia, supporting a population of approximately 5 million, and as the headquarter of the African Union hosts many transient visitors from diverse countries. There are two major branches of Akaki river flowing through the centre of Addis Ababa, the Little Akaki and the Big Akaki rivers. The Big Akaki River originates in the north-eastern region of Addis Ababa and passes through residential and commercial areas as well as by major hospitals and healthcare facilities of the city (Fig. 1a). The Little Akaki River originates in the north-western part of the city in the Gefersa reservoir and passes through a heavily industrial zone. The two rivers join at Aba-Samuel reservoir and form one of the biggest tributaries of the Awash River. The Akaki river system runs through a densely populated area where households release untreated waste directly into the river. Several socio-economic activities such as health care facilities, markets, industries, diagnostic, and research laboratories as well as irrigation of agricultural land are situated along the Akaki river system and contribute with untreated effluent (EPHI 2017).

Sediment and water samples were obtained from five sampling points: Gefersa (GE), Mekanissa (MK), Batu (BA), Zewditu (ZE), and Aba-Samuel (AB), which were selected along both Akaki river systems (Fig. 1a). The sampling sites were selected to reflect different anthropogenic activities along the river in addition to residential input based on the previous surveillance by the Ethiopian Public Health Institute for physicochemical and indicator bacteria analysis (EPHI 2017). GE, a reservoir upstream of the Little Akaki River, is the main source of drinking water for Addis Ababa and is restricted for human and other anthropogenic activities. MK is in an irrigation and residential area where effluent from households and agricultural activities are directly released into the river system. In addition, the water at this site is used for car washing and the wastewater is returned to the river untreated. BA is in an industry-dominated area and irrigation zone of the Little Akaki river. The industries in this area include leather and dye factories that release waste directly into the river system. Waste and run-off from irrigation farms also enter the river at this site. ZE is in the Big Akaki river located along a residential area with healthcare facilities. Most referral hospitals and public health research laboratories are located along the Big Akaki river and they potentially release their effluent to the river. AB is the downstream reservoir where both Akaki rivers converge. The water from the AB reservoir flows through an electric power plant before it enters the Awash River. The reservoir is restricted to human activities; however, just upstream are areas dominated by small-scale irrigation farms that release wastewater to the river (Fig. 1a). All three sites (MK, BA, ZE) have a higher residential population along the river than either GE or AB and only one rudimentary wastewater treatment plant is located between BA and AB sampling sites.

### Sample collection and DNA extraction

Sediment and water samples were collected from five locations along the Akaki river during the dry season (January) in 2020 and divided for separate DNA extractions. For comparison, the sediment and water samples for each site were obtained at the same time. Water samples were collected in sterile 1-L bottles from 15–20 cm below the water surface in the flow of the river. Sediment samples were collected in sterile polypropylene centrifuge tubes using a sediment sampler. The collected samples were transported on ice to Armauer Hansen Research Institute (AHRI) for same-day processing.

For DNA extraction from water, 50 ml was filtered through a 0.22-μm isopore polycarbonate filter (Merck Millipore, Ireland), and the filters were transferred to a PowerWater DNA bead tube before being stored at − 20℃ until DNA extraction. Total DNA was extracted from the filters using Qiagen DNeasy PowerWater Kit (Qiagen, USA) according to the manufacturer’s instructions. The kit has better extraction efficiency to extract DNA from water (Brandt and Albertsen 2018).

The sediments were stored at − 20 °C overnight and DNA was extracted the next day. Total DNA was extracted from 500 mg wet weight of sediments using the FastDNA SPIN Kit (MP Biomedicals, USA) according to the manufacturers’ instructions. The FastDNA SPIN Kit was selected for its high quantity and efficiency extraction of DNA from soil matrix (Dineen et al. 2010). Briefly, the samples were homogenized by bead beating in lysis buffer using the FastPrep instrument (MP Biomedicals, USA) at 6.0 m/s for 40 s, and the DNA was extracted using a spin column after protein precipitation, eluted, and stored at − 20 °C.

Three DNA replicate samples were extracted from each site and sample type and pooled for further analysis. The quality of DNA was checked with 0.8% agarose gel electrophoresis. The concentration and purity based on 260/280 and 260/230 ratios of the DNA measured using a NanoDrop 2000 Spectrophotometer (DeNovix, USA) was ≥ 1.8 and ≥ 2.0, respectively. The DNA was stored at − 20 °C until it was shipped to Örebro University, Sweden, on dry ice to prevent degradation.

### Detection and quantification of ARGs and bacteria

Microbial DNA qPCR arrays (QIAGEN, Sweden) were used to determine the diversity and abundance of ARGs and clinically relevant bacteria. Antibiotic resistance genes DNA qPCR array (Qiagen, Sweden, BAID-1901ZRA) and the water and sepsis DNA qPCR arrays (Qiagen, Sweden, BAID-1405Z and BAID-1903Z) were used for the detection of ARGs and bacterial pathogens according to the manufacturer’s instructions. The kits were designed to detect species specific bacterial and fungal ribosomal rRNA genes or selected ARGs using primers that were validated by the manufacturer using synthetic templates. A no-template control (NTC) was run for each set of arrays to establish the background CT values and as a quality control for contamination. NTC was also used to establish the lower cycle threshold value for positive calls (ΔCT ≥ 6), and the upper CT value for negative calls (ΔCT < 3) (Khan et al. 2019). PCR-positive control (PPC) was used to determine the presence of potential PCR inhibitors in the samples. Each array also contained two pan bacterial primers for total bacterial DNA as a quality check and normalization for relative quantification. The reactions were conducted in a final volume of 25 µl containing Microbial qPCR Master mix and 10 ng template DNA per reaction. The qPCR thermocycling conditions for SYBR Green consisted of an initial PCR activation step for 10 min at 95 °C followed by 40 cycles of 95 ºC for 15-s denaturation and 60 °C for 2-min annealing and extension in a CFX96™ Real-Time system (Bio-Rad Laboratories, Canada) according to the manufacturer’s instructions.

### Statistical analysis

The presence or absence determination for ARGs and bacteria was analyzed by the ΔCT (ΔCT = CT_Test sample_ − CT_NTC_) method using the data analysis template in Excel software provided by the manufacturer (Qiagen). For relative profiling, the ΔΔCT method (2^−ΔCT Test sample^/2^−ΔCT NTC^) was used to calculate the fold change of each gene and bacterial species using total bacterial genomic DNA of the upstream river as a control for normalization. A heat map was constructed by GraphPad Prism version 8.3 for Windows (GraphPad Software, La Jolla California, USA). Principal coordinate analysis (PCA) was performed to evaluate differences in ARGs and bacterial community profiles among sampling sites based on the Bray–Curtis distance of ARGs and bacterial relative abundance. A Pearson correlation matrix (Pearson) was used to determine the correlation between ARGs and the bacterial community and *P* < 0.05 was considered significant. The correlation plot between ARG profiles and bacterial communities was determined by Redundancy analysis using Canoco 5.0 software package (https://canoco.software.informer.com/5.0/).

## Results

### Greater diversity and abundance of ARGs in urban impacted sites of Akaki river water

**Fig. 2:**
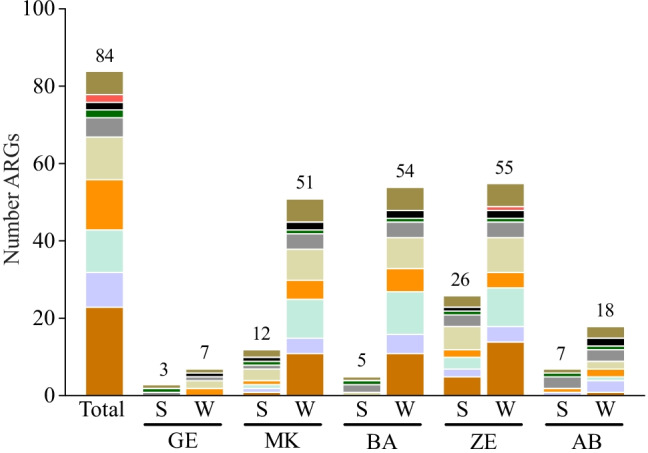
The antibiotic resistance genes detected from Akaki river sediment (S) and water (W) in Addis Ababa, Ethiopia. Sediment and water samples were collected from five sampling points along Little Akaki (GE, MK, and BA), Big Akaki (ZE), and a downstream reservoir (AB). MDR indicates multidrug resistance efflux pump genes and macrolide L. S. resistance indicates macrolide, lincosamide, and streptogramin resistance. Total indicates the total numbers of antibiotic resistance genes tested

In the present study, a total of 84 clinically relevant ARGs associated with resistance to β-lactams, aminoglycosides, fluoroquinolones, macrolides, and vancomycin, as well as genes associated with multidrug resistance and tetracycline efflux pumps, were analyzed from surface water collected from 5 different sites in the Akaki river. All sites were found to be positive for β-lactam, fluoroquinolone, aminoglycoside, macrolide, and tetracycline resistance genes at the class level. Spatial variation was observed for the diversity of ARGs among sampling locations. Fewer resistance genes were detected in the farthest site upstream, the Gefersa reservoir (GE), which is the main source of drinking water and where the Little Akaki river originates, as well as the downstream sampling point, the Aba-Samuel reservoir (AB), where both Little and Big Akaki rivers form a large tributary (Fig. 2). None of the multidrug resistance efflux pumps, class A, B, and C β-lactamases, vancomycin, and erythromycin resistance gene were detected in either of the upstream (GE) or downstream (AB) reservoirs. Only *bla*_OXA-10_, *aac(6)-Ib-cr*, *aadA1*, *mefA*, *tetA*, and *qnrS* were persistent in all sampling sites but in lower amounts in the furthest upstream and downstream sites. Unique resistance genes were detected in several sampling sites. For instance, vancomycin resistance gene (*vanB*), *bla*_KPC_, a *bla*_PER_ variant (*bla*_PER-2 group_), *bla*_SHV_ variant (*bla*_SHV(238 G240K)_), and *qepA* were exclusively detected in ZE, whereas three β-lactamase-encoding genes (*bla*_NDM_, *bla*_CFE-1_, and *bla*_OXA-23_) were detected only in BA just downstream of an industrial area. Two carbapenemase-encoding genes (*bla*_IMP-2_ and *bla*_IMP-5_) were only detected downstream of the river. Similar to the β-lactamase genes, the middle catchment of the river contained most aminoglycoside resistance genes.

**Fig. 3:**
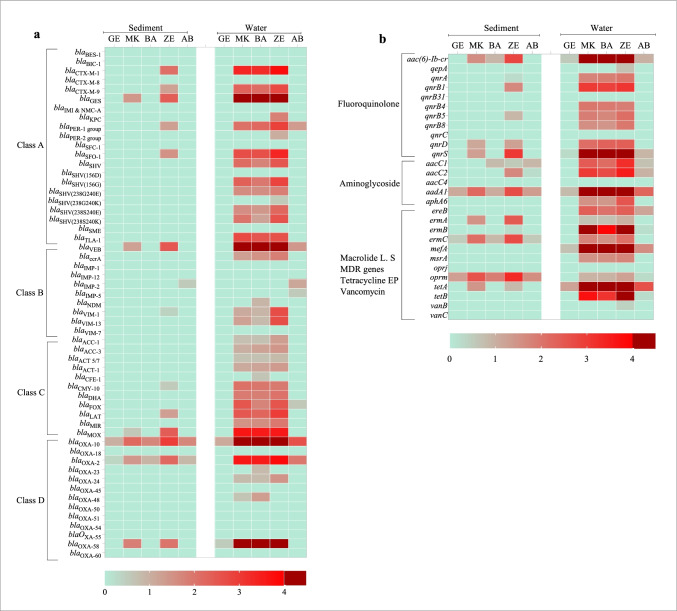
Relative abundance of **a** β-lactamase genes and **b** aminoglycoside, fluoroquinolone, macrolide, MDR efflux pump, tetracycline efflux pump, and vancomycin resistance genes in bacterial communities of Akaki river sediment and water samples. Genes grouped by antibiotic class are demarcated by black line on the left side. Green indicates the absence and red indicates the presence of genes. The gradient of the red reflects the different abundance of resistance gene based on fold change. The dark red indicates high abundance (high fold change) of a particular gene. The sites are Gefersa (GE), Mekanissa (MK), and Batu (BA) from Little Akaki river, and Zewditu (ZE) from Big Akaki river and both rivers join in the Aba-Samuel (AB) reservoir

Not only the diversity of ARGs but also their abundance demonstrated spatial variation. The relative abundance of ARGs was the highest in the three anthropogenic impacted sites (MK, BA, and ZE) and the lowest in the locations furthest upstream (GE) and downstream (AB) in the river. The abundance of *tetA* and *bla*_OXA-10_ genes was highest of all the genes in all sites. The fold change in the middle catchment of the river also showed some variations (Fig. 3). In general, both the number (Fig. 2) and abundance (Fig. 3) of ARGs detected in the upstream and downstream river water were much lower than the other three sites. For instance, only 18% of fluoroquinolone and 3.6% of β-lactam resistance genes were detected upstream of the Little Akaki river.

The study investigated 55 β-lactamase genes belonging to four sub-classes (A, B, C, and D) and found that their distribution and diversity showed spatial variation among sampling sites. A majority (69%) of the tested β-lactamase-encoding genes were detected in the river water. The proportion of β-lactam resistance genes at the sampling points was 1.9%, 28.8%, 31.7%, 30.8%, and 6.7% for GE, MK, BA, ZE, and AB, respectively (Fig. 3a). The Gefersa reservoir, upstream of the Little Akaki river, contained only two β-lactamase-encoding genes (*bla*_OXA-58_ and *bla*_OXA10_), both from class D. Most of the β-lactamase genes were detected in three sites (MK, BA, and ZE) nearest the industries, healthcare facilities and agricultural activities. In some sites, unique β-lactamase genes were detected. None of the class A, B, and C β-lactam resistance genes were observed upstream of the river. All tested class C β-lactamase genes were detected in the river, and except for *bla*_FOX_, all were near the industrial, hospitals and agricultural regions of the rivers, MK, BA, and ZE. None of *bla*_BES-1_, *bla*_BIC-1_, *bla*_CTX-M-8_, *bla*_IMI&NMC-A_, *bla*_SFC-1_, *bla*_SHV(156D)_, *bla*_SME_, *bla*_IMP-1_, *bla*_IMP-12_, *bla*_VIM-7_, *bla*_OXA-18_, *bla*_OXA-45_, *bla*_OXA-50_, *bla*_OXA-51_, *bla*_OXA-54_, *bla*_OXA-55_, and *bla*_OXA-60_ were detected in any of the sampling sites (Fig. 3a).

The presence and abundance of five major aminoglycoside resistant genes (*aacC1*, *aacC2*, *aacC4*, *aadA1*, and *aphA6*) were determined, of which *aadA1* was consistently observed at all sampling sites (Fig. 3b). Four (*aacC1*, *aacC2*, *aadA1*, and *aphA6*) were detected in three sites (MK, BA, and ZE). The middle catchment of the river was where most fluoroquinolone resistance genes were detected, while the upstream and downstream of the river contained only two fluoroquinolone-resistant genes [*qnrS* and *aac*(*6*)*-Ib-cr*]. Of the 5 macrolide resistance genes (*ermA*, *ermB*, *ermC*, *mefA*, and *msrA*) assessed, only *mefA* was found at all sampling sites. The three anthropogenically impacted sampling sites (MK, BA, and ZE) contained all tested macrolide resistance genes (Figs. 2 and 3b) and of the two multidrug efflux pump genes (*oprJ* and *oprM*), only *oprM* was detected in four sites (MK, BA, ZE, and AB). Of the two tetracycline resistance genes (*tetA* and *tetB*) analyzed, *tetA* was persistently detected in all locations, while *tetB* was detected in four sites (MK, BA, ZE, and AB). One vancomycin gene (*vanB*) was detected in the hospital site and none of the sampling sites contained *vanC* (Fig. 3b).

### Akaki river sediments have lower diversity and abundance of ARGs

Sediment samples were collected from all five sites in the river and assessed for the presence and relative abundance of ARGs as for the water samples. Fewer ARGs were detected in Akaki River sediment samples than in the respective waters (Fig. 2a). Two β-lactam (*bla*_OXA-10_ and *bla*_OXA-2_), 1 aminoglycoside (*aadA1*), and 1 macrolide (*ermC*) resistance genes and 1 multidrug efflux pump (*oprM*) were detected in all sediment samples. A relatively higher number of ARGs were detected at ZE compared to other sites. For instance, only two β-lactamase genes were detected in BA, whereas 13 β-lactamase genes were detected in ZE (Fig. 3a). Not only the β-lactamase genes, but also other resistance classes showed spatial variations between sampling sites. Unique genes detected at ZE include *bla*_CTX-M1_, *bla*_CTX-M-9_, *bla*_PER-1_, *bla*_SFO-1_, *bla*_VIM-1_, *bla*_CMY-10_, *bla*_LAT_, *qnrB1*, *qnrB5*, and *aaC2*. Like the water samples, *bla*_IMP-2_ was only detected downstream of the river (AB) (Fig. 3a).

**Fig. 4:**
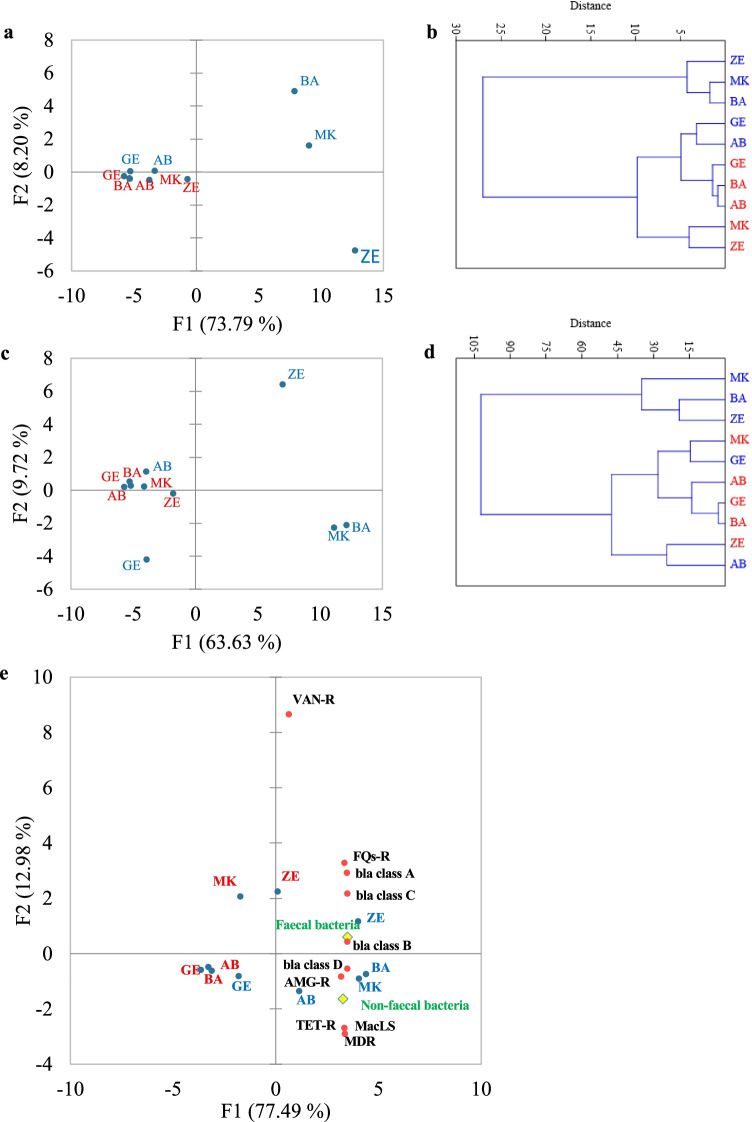
Correlation matrix biplots. **a** Score plot showing the distribution of ARGs in Akaki river sediment. **b** Agglomerative hierarchical clustering computation of ARG levels in Akaki river sediment (red) and water (blue). **c** Score plot showing the distribution of clinically relevant bacteria profiles of GE, MK, BA, ZE, and AB sediment (red) and water (blue). **d** Agglomerative hierarchical clustering computation of clinically relevant bacteria levels in Akaki river sediment and water. **e** Score plot showing the distribution of ARGs at class levels, fecal and non-fecal bacteria in Akaki river sediment (red) and water (blue). The correlation analysis was based on the fold changes of ARGs and clinically relevant bacteria profile in different samples. Abbreviations: VAN-R, vancomycin resistance; FQs-R, fluoroquinolone resistance; AMG-R, aminoglycoside resistance; TET-R, tetracycline resistance; MacLS, macrolide L. S. resistance; MDR, multidrug resistance efflux pump; bla class A, beta-lactamase class A; bla class B, beta-lactamase class B; bla class C, beta-lactamase class C; bla class D, beta-lactamase class D

ARGs encoding for aminoglycoside, fluroquinolone, and macrolide resistance were detected in greater numbers than the β-lactamase-encoding genes in most sites. The sediment from BA, AB, and GE did not contain class A β-lactamase-encoding genes; however, a relatively greater number were detected in ZE site. Fluroquinolone and aminoglycoside resistance genes were not detected in furthest upstream or downstream sites. As for specific β-lactamase-encoding genes, *bla*_OXA-10_ was the most abundant and the highest abundance was observed in ZE sediments (Fig. 3). Similarly, the highest abundance of other classes of resistance genes was observed in ZE. The correlation analysis showed a significant correlation between the number of ARGs detected in the sediment collected from ZE with waters in ZE (*r* = 0.821), MK (*r* = 0.801), and BA(*r* = 0.804), although the association is not very strong. There was also a significant association between sediment collected from ZE and MK sites (*r* = 0.887) (Fig. 4a, b).

### Clinically important bacteria in water and sediments of the Akaki River

**Fig. 5:**
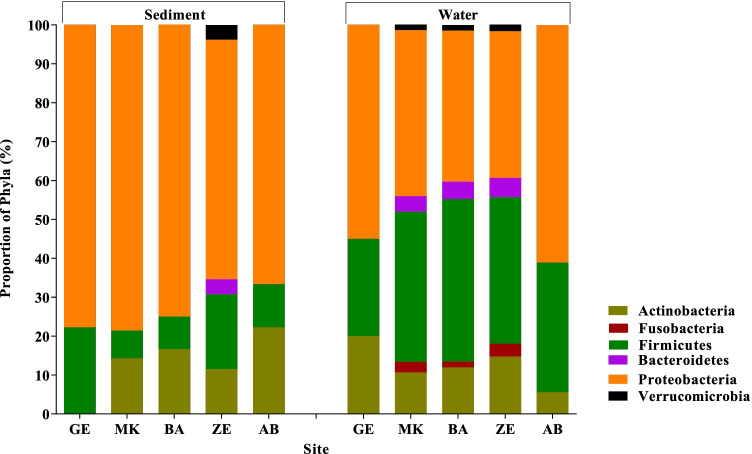
Distribution of clinically relevant bacteria at the phyla level detected by qPCR from Akaki river sediment and water samples. The sites are Gefersa (GE), Mekanissa (MK), and Batu (BA) from Little Akaki river, and Zewditu (ZE) from Big Akaki river and both rivers join in the Aba-Samuel (AB) reservoir

**Fig. 6:**
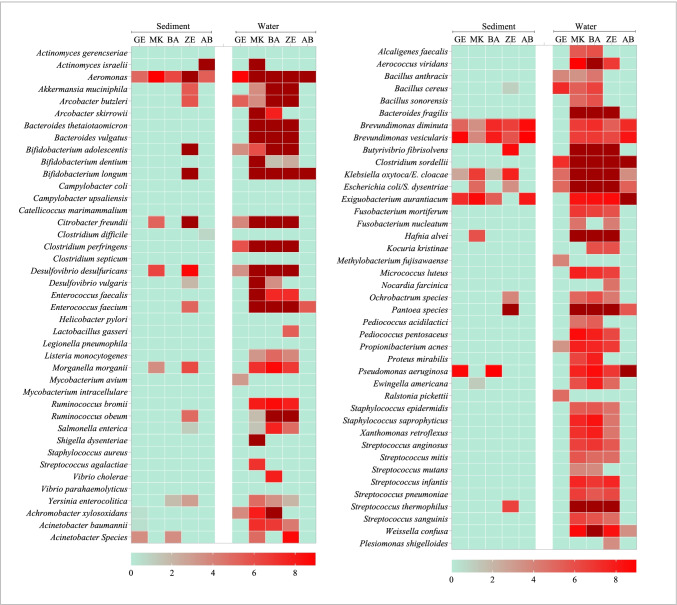
Relative abundance of clinically relevant bacteria from Akaki river water and sediment samples. The log fold change of each bacterial species was calculated using ΔΔCT method. Green color indicates the absence and red color indicates the presence of genes. Gradient of the red color reflects increasing abundance based on the difference in the fold change of detection of bacterial DNA. The dark red indicates a high abundance based on high fold change of a particular bacterial species

Using microbial DNA qPCR arrays, we determined the diversity, distribution, and relative abundance of clinically important bacteria in the Akaki river (Table 1). The bacteria identified in the Akaki river were grouped into six phyla: Actinobacteria, Fusobacteria, Firmicutes, Bacteroidetes, Proteobacteria, and Verrucomicrobia. The most abundant phylum identified in the river water samples was Proteobacteria followed by Firmicutes, Actinobacteria, Bacteroidetes, Fusobacteria, and Verrucomicrobia. The distribution at the phyla level showed a spatial variation between sampling sites. Three phyla (Actinobacteria, Firmicutes, and Proteobacteria) were persistently detected in all sampling sites. All six phyla (Actinobacteria, Fusobacteria, Firmicutes, Bacteroidetes, Proteobacteria, and Verrucomicrobia) were detected in the middle catchment of the rivers (MK, BA, and ZE); however, Fusobacteria, Verrucomicrobia, and Bacteroidetes were not identified in the upstream and downstream of the river (GE and AB) (Fig. 5). Distribution of fecal bacteria has also shown spatial variation between sampling sites. In the furthest upstream, only 25% of bacteria were of fecal origin; whereas in the three anthropogenically impacted sites, 43–48% of identified bacteria were of fecal origin and the highest was observed in ZE. Similar trends were observed in the sediment sample in which 60% of the bacterial isolates in ZE were of fecal origin and none were detected in sediment from AB, the furthest downstream of the river. The findings showed that MK and ZE were highly contaminated by fecal bacteria, and this could be a plausible cause for the increased abundance of ARGs (Fig. 1b). The major bacterial species detected in all sampling sites were *Aeromonas* species, *Clostridium sordellii*, *Klebsiella oxytoca*, and *Escherichia coli*/*Shigella dysenteriae* (Supplementary Table S1). The bacterial richness and diversity were highest in MK, BA, and ZE while fewer bacterial species were identified upstream and downstream of the river (Fig. 6). Multivariate analysis clustered MK and BA together and GE and AB together (Fig. 4c). Site-specific bacteria were also detected in the middle catchment of the river. For instance, *Vibrio cholerae* was identified only in BA sampling site. Most of the bacteria tested were not detected in the sediment samples. Two phyla (Firmicutes and Proteobacteria) were persistently detected in all sampling sites (Fig. 5). At the species level, *Aeromonas* species, *Brevundimonas diminuta*, and *Brevundimonas vesicularis* were persistently identified in all sampling sites. The sediment sample collected from the hospital site (ZE) contained a relatively higher number of bacteria.

**Table 1:**
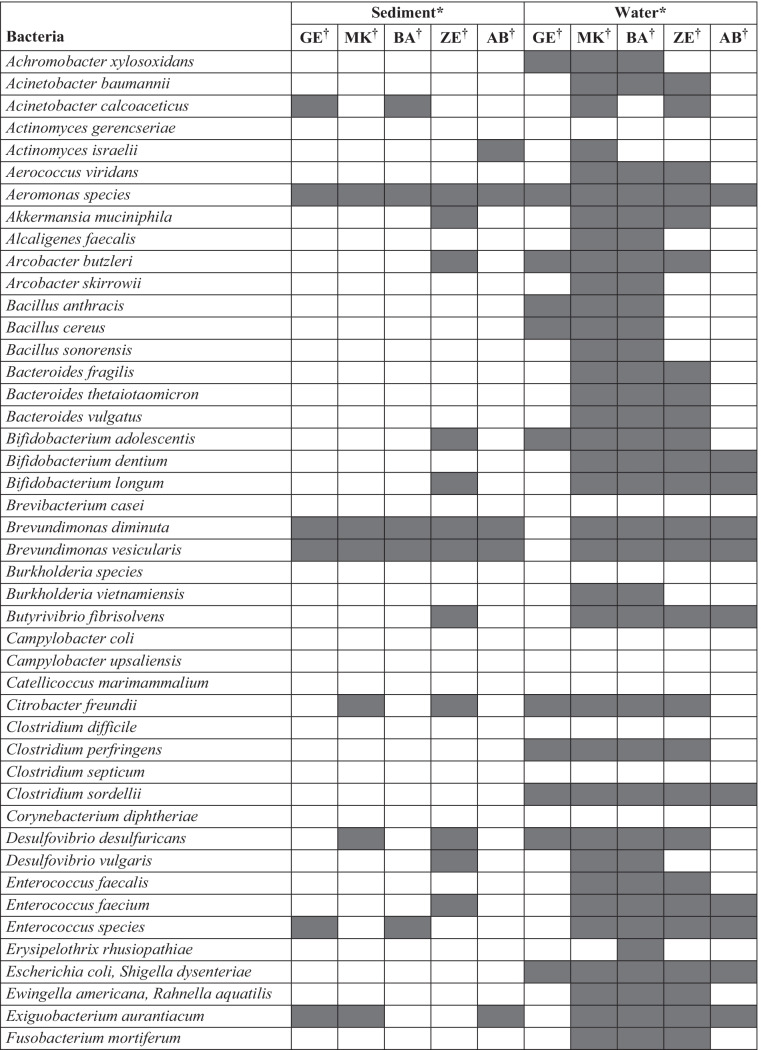

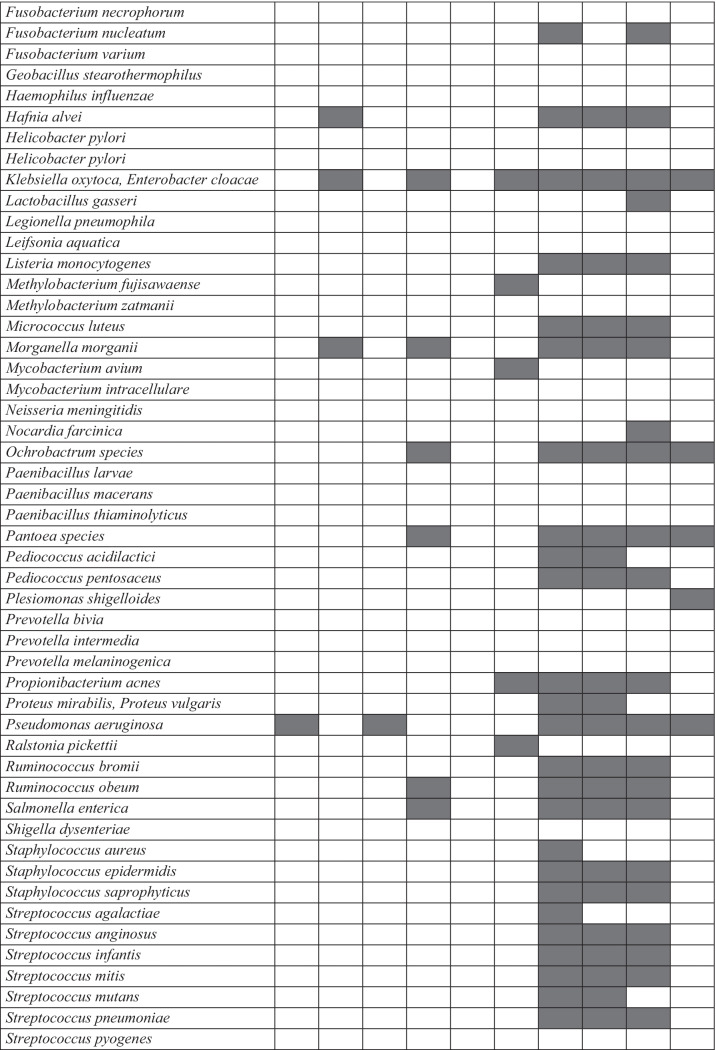
The presence of clinically relevant bacteria identified in sediments and water at different locations in the Akaki river

Shaded boxes indicate presence of bacteria*.* *GE* Gefersa, *MK* Mekanissa, *BA* Batu, *ZE* Zewditu, *AB* Aba-Samuel

### Diversity and abundance of ARGs correlated with the abundance of bacterial phyla and fecal bacteria

A significant correlation was observed between bacterial abundance at the phyla level and ARGs. A correlation was observed in Bacteroidetes and Firmicutes with β-lactamases and erythromycin resistance genes; Fusobacteria, Proteobacteria, Firmicutes, and Bacteroidetes with aminoglycosides, macrolides, and multidrug resistance efflux pumps; and Fusobacterial, Bacteroidetes and Firmicutes with fluoroquinolone and tetracycline efflux pump. In addition to the phylum level, redundancy analysis with Pearson correlation coefficient was performed to determine the relationship between bacterial species and ARGs. The bacterial richness was positively correlated with the abundance and the diversity of ARGs in the three sites (MK, BA, and ZE). The spatial difference of bacterial diversity and their abundance in each site also resulted in the presence of unique antibiotic resistance. In the redundancy analysis, the fold change of some bacteria was significantly correlated with the fold change of ARGs. For instance, the majority of the ARGs were positively correlated with *Bacteroides vulgatus*, *Bacteroides thetaiotaomicron*, *Clostridium perfringens*, *Enterococcus faecium*, *Lactobacillus gasseri*, *Aeromonas enteropelogenes*, and *Micrococcus luteus* (Supplementary Table S2). The upstream and the downstream sites had fewer bacterial compositions with a low level that resulted in few ARGs compared to other sites. Strong correlation was observed between fecal bacteria and majority of the resistance classes, with a correlation coefficient (*r*) of 0.89, 0.84, 0.93, and 0.91 for class A β-lactamase, class B β-lactamase, class C β-lactamase, class D β-lactamase and genes conferring resistance to fluoroquinolones, respectively (Fig. 4c). ARG classes significantly associated with non-fecal bacteria were class D β-lactamase (*r* = 0.90) and genes providing resistance to macrolide, lincosamide, and streptogramin (MacLS) (*r* = 0.92). The sediment samples collected from the four sites (AB, MK, BA, and AB) clustered together, whereas ZE did not cluster with any other site. The increased diversity and abundance of ARGs in the sediment collected from ZE linked with the presence of more fecal origin bacteria than other samples (Fig. 4e).

## Discussion

ARGs are emerging environmental pollutants because of the continual discharge of both ARB, and pharmaceutical and chemical pollutants into the environment (Yang et al. 2018). The diversity and the level of ARGs are related to the presence of ARB in the both sediments and waters of aquatic environments (Baker-Austin et al. 2006). In addition to industrial and hospital waste, a major contributing factor to the increasing levels of ARGs in the environment includes fecal pollution in urban regions (Newton et al. 2013; Karkman et al. 2019). Rivers in Addis Ababa are severely impacted by direct discharge of untreated effluents from the community and industries (Hiruy et al. 2022). As in many regions, the spread of ARB in clinical settings is well documented in Ethiopia; however, the community acquired resistance, as well as the contribution of environmental factors, is not well known (Moges et al. 2014). It is therefore critical to better understand the distribution, diversity, and level of ARGs and associated clinically important bacteria in the aquatic environment to improve management of these microbial pollutants.

Aquatic sediments act as reservoirs of ARB and ARGs, mainly in rivers receiving effluents containing bacteria and drug residues from sewage (Heß et al. 2018). However, the Akaki river sediments contained significantly fewer ARGs with some spatial variations between sampling sites when compared to the water compartment. One possible explanation for the low abundance and diversity of ARGs in the sediment relative to the water may be that the different ARGs have different bacterial hosts and these bacteria potentially have different distribution in the aquatic and sediment phases. In the present study, a lower bacterial composition and abundance was observed in the sediments than water phase. Most ARGs detected in the sediment were shared by the river water. This low prevalence of ARGs could be due to the absence of ARB in sediment as observed with both a low bacterial diversity and density in the present study. Although there was a relatively higher diversity and level of ARGs in sediment collected from the site near hospitals (ZE), they were still much lower than in the water phase. This could be due to the nature of the sediments as well as the source of the pollution. The sediment near the hospital site was characterized as sandy/clay and it was reported that clay sediments facilitate the persistence of resistant pathogens due to their high adsorption capacity for ARGs (Guo et al. 2020; Mao et al. 2014). Our results are in line with Zhang et al*.* (Zhang et al. 2021) reporting greater diversity and abundance of ARGs in a sediment collected from hospital and animal farm sites than those mainstream sites of the Maozhou river, China. Effluents from hospitals may contain pathogenic ARB that settle to the bottom of the river as it flows more slowly compared to the Little Akaki river. However, our results are in contrast to other reports that found comparable diversity and level of ARGs in both sediments and water of streams and lakes (Calero-Cáceres et al. 2017; Berglund et al. 2015; Allen et al. 2010). Fewer ARGs in the sites farthest upstream and downstream are comparable with the report in the Liaohe River Basin, China (Na et al. 2021), that showed that significantly lower ARGs were detected in rural sites than urban impacted sites. Using different DNA extraction kits for the sediments and water may be considered a potential limitation; however, since the protocols were optimized for the respective matrix, this is unlikely the cause of the large difference between the sediments and water. The conflicting reports suggest that it is yet unclear what factors are important for determining the abundance of ARGs in sediments relative to the respective waters and this is important for future management of antibiotic resistance in the environment.

Relatively greater numbers of the tested genes encoding for aminoglycoside, fluoroquinolone, and macrolide resistance were detected in the sediments than other resistance classes. These antibiotics are man-made (Adachi et al. 2013) or semisynthetic (Abdul-Mutakabbir et al. 2019) and are more persistent in the environment providing greater binding potential to sediments. Antibiotics and other pollutants may also increase the selection pressure on indigenous bacterial populations, enriching the resistant species (Kerrigan et al. 2018). Reports have shown that ARGs present in human pathogens have been detected in environments with no known history of antibiotic exposure or human activities, thus it is likely that native bacteria carry resistance determinants (Allen et al. 2010). In addition, bacteria associated with sediments are often within biofilms that promote genetic exchange due to higher bacterial density (Ding and He 2010). Despite the low levels, the presence of ARGs in the sediment indicates that the natural environment can act as a reservoir for ARGs.

ARGs were detected in all sediment samples evaluated; however, abundance was higher in the ZE site. Our results showed a correlation between the abundance of fecal bacteria and ARGs in sediments collected from ZE (F1 77.49% of the variation) and had a strong linear relationship with genes encoding for fluoroquinolone, and class A, B, and C β-lactamase-encoding genes. Previously, it has been reported that fecal pollution drives the increasing diversity and abundance of ARGs in the environment (Karkman et al. 2019). These variations could be explained by the different types of anthropogenic activities at the sampling locations, including healthcare facilities and households in ZE site, which are the main contributors to the ARGs in the environment. Studies have reported that the levels of ARGs in sediments close to urban settlements are higher than in environments with no history of community association or anthropogenic activities (Berglund et al. 2015). While the presence of ARGs in the sediment of the Akaki river is a concern, as they can be released into the water phase, the low levels detected in the present study are reassuring. Thus, the immediate concern should be the water compartment, having a much higher level of ARGs and clinically relevant bacteria.

In the current study, we found β-lactam resistance genes were more widely distributed in the river water than the sediments. These genes are frequently plasmid encoded and easily transferred within bacterial communities, contributing to their rapid dissemination (Ceccarelli et al. 2019). However, the distribution significantly varied between sampling sites. The Gefersa (GE) reservoir located outside of Addis Ababa, upstream of the Little Akaki river, contained significantly fewer β-lactamase-encoding genes, with only *bla*_OXA-10_ and *bla*_OXA-58_ detected. The *bla*_OXA-10_ is commonly reported in *Pseudomonas aeruginosa* isolates; however, it has also been found in Enterobacteriaceae (Maurya et al. 2017). Most of *bla*_OXA-10_-derived ESBLs confer resistance to third-generation cephalosporin and aztreonam (Naas et al. 2008). Since the levels of *bla*_OXA-10_ are very low upstream of the river, it could be that it is intrinsically present in the bacterial community as previously reported (Yang et al. 2019) or due to a low-level contamination by wild birds (Darwich et al. 2019). The gene is consistently present in sediment and water phases suggesting a continual release of the gene from sediment to water is possible. Anthropogenically impacted sites are more prone to pollution by bacteria harboring *bla*_OXA-10_ and thus exhibiting higher levels as seen in the Little (MK, BA) and Big (ZE) Akaki river sites. High level of urbanization can contribute to the spatial differences as evidenced by the increased number of fecal bacteria detected in the middle catchment of the river (MK, BA, and ZE). The β-lactamase-encoding genes’ distribution pattern of the three sites (MK, BA, and ZE) was more closely correlated, suggesting that anthropogenic activities within the city were the major contributors to this class of ARGs in the river. Furthermore, MK site is in proximity of an irrigation system with fertilizers and pesticides which when discharged into the river could trigger the persistence of resistance genes since bacteria resistant to pesticides are also often resistant to antibiotics (Ramakrishnan et al. 2019; Xie et al. 2022). This further suggests that anthropogenic activities contribute to ARGs in the river. The result is comparable with a previous study that showed a positive correlation between ARGs and anthropogenic activities (Jiang et al. 2018).

The New Delhi metallo-β-lactamase 1 (*bla*_NDM-1_) gene was detected only in BA (Little Akaki river). The gene is normally located on a self-transmissible plasmid that can be easily transferred between the same or different species. Bacteria such as *K. pneumonia*, *E. coli*, *Acinetobacter* sp., and *Enterobacter* sp. carry the gene. Discharge from hospitals frequently contain bacteria that are positive to *bla*_NDM-1_ (Islam et al. 2017); however, in this study, it was found in a site not associated with hospitals suggesting it may either be present in the community or farm animals. Poultry farming at this site may contribute to the persistence of the *bla*_NDM-1_ gene, as poultry farms are hotspots for the spread of resistant pathogens (Dandachi et al. 2018). It was recently reported that *bla*_NDM-1_ was detected in bacteria isolated from rivers in India (Ranjan et al. 2021). The presence of genes that confer resistance to last resort antibiotics in the environment highlights the need of increased measures to control the spread of resistant pathogens.

Healthcare facilities and their effluent are one of the major sources of ARG transmission to the environment (Devarajan et al. 2016). The hospital site (ZE) in the current study contained unique β-lactamase-encoding genes that are potentially in untreated wastewater discharged from the hospitals and community (Fig. 3). *bla*_KPC_ is encoded on transferable plasmids and bacteria that have KPC enzymes confers resistance to most β-lactam antibiotics (Anderson et al. 2007). Previously, the gene has been reported from hospital effluents in Peru (Soriano-Moreno et al. 2021). There was a relatively higher abundance of resistance genes in the site close to the hospitals suggesting that the hospitals discharged untreated wastes directly to the Akaki river or its tributaries. In both sediment and water samples, it was found that the presence of fecal bacteria positively correlated with increased abundance of ARGs, primarily with gene encoding for fluroquinolone resistance and class A, B, and C β-lactamases. The difference in the abundance of ARGs in urban impacted sites is associated with the varying levels of fecal pollution. Most fecal bacteria tested were detected in ZE, both in the sediment and water. Our results are in line with other reports that have shown effluents from hospitals result in an increased abundance of ARGs in their receiving river (Khan et al. 2019). The sediments collected from the ZE site contained higher diversity and abundance of ARGs than the other sites evaluated, and this could be related to higher discharge from hospitals, diagnostic facilities, and fecal pollution. In addition, the abundance and diversity of bacterial communities also correlated with the increased abundance of ARGs (Khan et al. 2019). There was also an increased abundance of *A. baumannii*, which is intrinsically resistant to many antibiotics effective against Gram-negative bacteria (Manchanda et al. 2010). Effluent from hospitals together with fecal pollution in Addis Ababa is a major contributor to the spread of β-lactamase and carbapenemase-encoding genes and may pose a public health hazard to the population living near the Akaki river system.

Much fewer β-lactamase-encoding genes were detected downstream of the river. This could be a result of reduced or no selection pressure due to lower levels of pollution and increased dilution. Unique carbapenem resistance genes (*bla*_IMP-2_ and *bla*_IMP-5_) detected in the Aba-Samuel reservoir (AB) are more likely due to contamination from animals such as birds, since the site is far downstream of the highly urbanized and industrialized regions of the Akaki river systems. Birds are reported to have a significant role in the emergence and transmission of (Wang et al. 2021) and have been shown to have a significant number of carbapenem resistant bacteria among migratory birds in China (Liao et al. 2019). Other non-point sources of pollution upstream of AB could also contribute to the presence of these genes. The downstream of the Akaki river system which is devoid of human activity can also be associated with the dissemination of resistance genes especially those in the imipenem group.

Streptomycin 3″-adenylyltransferase-encoding gene, *aadA1*, is distributed in a wide range of bacteria including *Aeromonas* spp*.*, *Citrobacter* spp*.*, *Shigella* spp., (Zhang et al. 2009), and *A. baumannii* (Khoshnood et al. 2017). The persistence of the gene in all sampling sites in the current study is likely due to the persistence of the *Aeromonas* and *Citrobacter* species. *Aeromonas* was detected in the sediment and water samples of all sites with highest abundance at the hospital site and that may have resulted in the highest abundance of *aadA1* gene. The level of aminoglycoside resistance genes was highest in areas associated with hospitals and thus are likely due to hospital discharge containing ARB or residual antibiotics. A similar study of hospital discharge in Sweden has shown the highest levels of aminoglycoside resistance genes (Khan et al. 2019). The gentamycin resistance gene, *accC1*, is present in different bacterial communities in the environment (Zhang et al. 2009) and was persistent in all the sampling points except the Gefersa reservoir. The presence of aminoglycoside resistance genes reflects the wide use of aminoglycosides and the increase of resistant pathogens in the healthcare facilities of Addis Ababa.

Fluoroquinolone antibiotics are widely used because of their efficacy against both Gram-negative and Gram-positive bacteria (Schindler et al. 2017). The consumption of fluoroquinolones in Addis Ababa correlates with the emergence of resistance, as often seen in countries where consumption of fluoroquinolones is high and resistance is also high (Redgrave et al. 2014). In Ethiopia, fluoroquinolones are the second most prescribed antibiotics in healthcare facilities (Worku and Tewahido 2018) and a meta-analysis reported a significant prevalence of fluoroquinolone resistance in both Gram-positive and Gram-negative bacteria (Sisay et al. 2018). However, the role of the environment on the persistence and spread of fluoroquinolone resistance is not reported in Ethiopia. We determined the presence of 11 plasmid-mediated quinolone resistance genes and found *aac(6)-Ib-cr* and *qnrS* were detected in all sampling sites of the rivers. *Escherichia*, *Aeromonas*, and *Acinetobacter* are common bacteria in aquatic environments that develop resistance to fluoroquinolones and it is most frequently due to the *aac*(*6*′)-*1b-cr* gene. This gene is transferable and encodes for aminoglycoside acetyltransferase that neutralizes aminoglycosides and ciprofloxacin. The *qnrS* gene has been reported mostly from *Aeromonas* and *Vibrio* species isolated in an aquatic environments (Osinska et al. 2016; Poriel et al. 2012)*.* The high abundance of *qnrS* gene in the hospital site (ZE) is most likely due to the presence of human-derived pathogens from hospital discharge or fecal pollution from households. The gene encoding for the fluoroquinolone efflux pump, *qepA*, is primarily found in isolates from animal sources and is rarely reported from clinical isolates. Although efflux pumps are chromosomal encoded, *qepA* is a plasmid-mediated gene (Poriel et al. 2012) so it can be horizontally transferred to pathogenic bacteria. The occurrence of fluroquinolone resistance genes in the Akaki river system is associated with the wide use of fluoroquinolone and contamination of the environment since they are man-made antibiotics.

## Conclusions

The present study reports the abundance and diversity of ARGs and clinically relevant bacteria in both sediments and waters from different sites along the Akaki river in Ethiopia. Notably, the sediments had consistently much lower levels ARGs relative to the waters. The lactamase-encoding genes were lower in diversity and abundance in the sediment, whereas aminoglycoside and fluoroquinolone resistance genes were more enriched. Surprisingly, the water phase of the urban impacted aquatic environment, rather than the associated sediments, is potentially the main source of persistence and spread of ARGs and thus ARB in the Akaki river. The highest levels of ARGs were in the urban impacted sites with a range of anthropogenic activities, including agriculture, hospitals, industries, and highly populated residential areas that release untreated waste and contributed to the diversity and abundance of ARGs. Thus, the river becomes the reservoirs of major clinically relevant ARGs that can be potentially transmitted among environmental and pathogenic bacteria in the river and subsequently to the residential population, contributing to a public health risk. To effectively implement an antimicrobial stewardship program, the role of the environment in the generation and spread of ARGs must also be considered. This study highlights the importance of considering the aquatic environment in the antimicrobial resistance surveillance system in Ethiopia for effective control of the spread of antibiotic-resistant bacteria and resistance genes.

## Supplementary Information

Below is the link to the electronic supplementary material.

Supplementary file1 (XLSX 79 KB) Correlation between bacterial communities and antimicrobial resistance genes. Values in bold are different from 0 with a significance level alpha ≤0.05.

## Data Availability

The datasets used and/or analyzed during the current study are available from the corresponding author on reasonable request.
